# Quantifying the reduction of respiratory motion by mechanical ventilation with MRI for radiotherapy

**DOI:** 10.1186/s13014-022-02068-5

**Published:** 2022-05-21

**Authors:** Z. van Kesteren, J. K. Veldman, M. J. Parkes, M. F. Stevens, P. Balasupramaniam, J. G. van den Aardweg, G. van Tienhoven, A. Bel, I. W. E. M. van Dijk

**Affiliations:** 1 Department of Radiation Oncology, Amsterdam UMC Location University of Amsterdam, Meibergdreef 9, Amsterdam, The Netherlands; 2 Department of Anesthesiology, Amsterdam UMC Location University of Amsterdam, Meibergdreef 9, Amsterdam, The Netherlands; 3 Department of Anesthesiology, Amsterdam UMC Location Vrije Universiteit Amsterdam, De Boelelaan 1117, Amsterdam, The Netherlands; 4 Department of Pulmonology, Amsterdam UMC Location University of Amsterdam, Meibergdreef 9, Amsterdam, The Netherlands

**Keywords:** Radiotherapy, MRI, Respiratory motion, Mechanical ventilation, Breathing control

## Abstract

**Background:**

Due to respiratory motion, accurate radiotherapy delivery to thoracic and abdominal tumors is challenging. We aimed to quantify the ability of mechanical ventilation to reduce respiratory motion, by measuring diaphragm motion magnitudes in the same volunteers during free breathing (FB)*,* mechanically regularized breathing (RB) at 22 breaths per minute (brpm), variation in mean diaphragm position across multiple deep inspiration breath-holds (DIBH) and diaphragm drift during single prolonged breath-holds (PBH) in two MRI sessions.

**Methods:**

In two sessions, MRIs were acquired from fifteen healthy volunteers who were trained to be mechanically ventilated non-invasively We measured diaphragm motion amplitudes during FB and RB, the inter-quartile range (IQR) of the variation in average diaphragm position from one measurement over five consecutive DIBHs, and diaphragm cranial drift velocities during single PBHs from inhalation (PIBH) and exhalation (PEBH) breath-holds.

**Results:**

RB significantly reduced the respiratory motion amplitude by 39%, from median (range) 20.9 (10.6–41.9) mm during FB to 12.8 (6.2–23.8) mm. The median IQR for variation in average diaphragm position over multiple DIBHs was 4.2 (1.0–23.6) mm. During single PIBHs with a median duration of 7.1 (2.0–11.1) minutes, the median diaphragm cranial drift velocity was 3.0 (0.4–6.5) mm/minute. For PEBH, the median duration was 5.8 (1.8–10.2) minutes with 4.4 (1.8–15.1) mm/minute diaphragm drift velocity.

**Conclusions:**

Regularized breathing at a frequency of 22 brpm resulted in significantly smaller diaphragm motion amplitudes compared to free breathing. This would enable smaller treatment volumes in radiotherapy. Furthermore, prolonged breath-holding from inhalation and exhalation with median durations of six to seven minutes are feasible.

***Trial registration*:**

Medical Ethics Committee protocol NL.64693.018.18.

## Background

Due to respiratory motion, accurate radiotherapy to thoracic and upper abdominal tumors and targets is challenging. Respiratory motion management is recommended when organ or tumor motion magnitude exceeds 5 mm [1]. Different strategies to account for respiratory motion are applied in treatment planning and radiation delivery. In treatment planning, margins are added to the gross tumor volume (GTV) and the clinical target volume (CTV), resulting in a planning target volume (PTV) to which the radiation dose is prescribed. To account specifically for respiratory motion an internal target volume (ITV) may be applied. The ITV is defined as the union of the CTV in all phases of the respiratory cycle, resulting in a relatively large PTV [2]. Alternatively, only the mid-ventilation part of the cycle may be used to avoid large PTVs [3]. However, all margins beyond the CTV result in radiation exposure of normal tissues, increasing the risk of acute and late radiation-associated toxicity [4]. Furthermore, because of variation in breathing motion, an ITV based on a single planning four-dimensional computed tomography (4DCT) or four-dimensional magnetic resonance imaging (4DMRI) might not fit the target volume at time of treatment, potentially leading to target underdosing and further overdosing of healthy tissue [5].

Other strategies to account for respiratory motion include abdominal pressure, respiratory gating, and tumor tracking [6–8]. These may be combined with instructing patients to breathe shallowly or to hold their breath [9–13]. Multiple deep inspiratory breath-holds (DIBH) are clinically implemented when treating tumors in the thorax and upper abdomen. Typically up to twelve consecutive DIBHs of ~ 20–30 s with air are used per radiation fraction, with suitable recovery periods in between [11]. However, the effectiveness depends on patients’ compliance with instructions [8, 11]. Tumor and organ position can vary considerably between consecutive DIBHs, depending on the inhaled volume and fatigue [14]. Furthermore, residual motion occurs during all breath-holds. [14–17].

In intensive care, non-invasive mechanical ventilation of unsedated patients has been developed over the last decades [18]. More recently, non-invasive mechanical ventilation has been explored in radiotherapy [19–24]. Another strategy under investigation is the use of continuous positive airway pressure which is originally meant to treat patients with sleep apnea and patients with chronic obstructive pulmonary disease [25–28]. Alternatively, several forms of high frequency ventilation with or without anesthesia have been applied in surgery and radiotherapy to suppress respiratory motion in the thorax and upper abdomen [29–33]. Previous studies have reported on various breathing control strategies in different volunteer and patient cohorts, but no comparison between breathing control strategies, including DIBH and prolonged breath-holding (PBH; duration up to > 5 min) were conducted within the same cohort. We aimed to investigate the advantages of rhythmic mechanical ventilation with positive pressure in reducing diaphragm motion. Mechanical ventilation offers radiotherapy two means of reducing organ motion, first by regularized breathing [23, 24] (RB), and second by PBH achieved by combining ventilation with preoxygenation and induced hypocapnia [20, 21, 34]. We are the first to compare diaphragm motion during free breathing (FB), multiple DIBHs, RB at 22 breaths per minute (brpm), and single PBH, all repeatedly performed by each of the healthy volunteers and measured with MRI.

## Methods

### Volunteer population

With approval of the medical ethics committee of the Amsterdam Medical Center (NL.64693.018.18), and after given written informed consent, 18 healthy volunteers enrolled in this study. They had no previous experience with non-invasive mechanical ventilation. Breathing control using mechanical ventilation for unsedated volunteers is feasible after proper training and volunteer preparation [20, 34].

### Training, volunteer preparation and safety

Volunteers were trained in two sessions to feel safe and comfortable being ventilated through a disposable face mask covering and sealing the mouth and nose. The mask was connected to a Hamilton MR1 mechanical ventilator (Hamilton Medical AG, Bonaduz, Switzerland) by a coaxial tubing, which enabled regularization of breathing and hyperventilation [20–22, 34]. Hyperventilation in 60% O_2_ induced hypocapnia to halve volunteers’ carbon dioxide (CO_2_) levels to 20 mmHg to enable a single PBH. Training and MRI sessions took place in supine position while measuring systolic and diastolic blood pressure (sBP and dBP), oxygen saturation (SpO_2_), heart rate, end-expiratory partial pressure of carbon dioxide (PETCO_2_), and airway pressure. The volunteer held their breath as long as they could, unless terminated by the investigator if the following occurred: sBP < 70 mmHg or > 180 mmHg, SpO_2_ < 94%, heart rate < 40 or > 130 beats per minute [21].

**Fig. 1:**
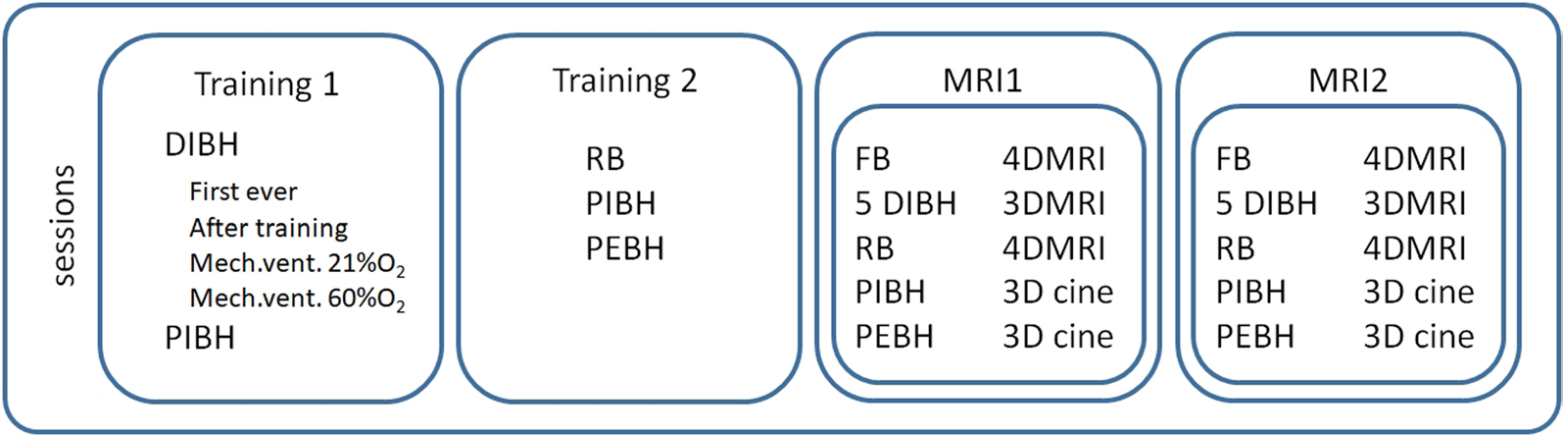
Overview of training and MRI acquisition sessions. Training 1 & 2 accustomed the volunteer to deep inspiration breath-holds (DIBH) with and without mechanical ventilation (Mech.vent.), regularized breathing (RB), prolonged breath-hold from inhalation (PIBH), and prolonged breath-hold from partial exhalation (PEBH). During MRI1 and MRI2, volunteers were scanned throughout FB and the various breathing control strategies. Imaging was either 4DMRI, 3DMRI or 3D cine-MRI (3D cine) as required

Figure 1 shows the training schedule. The first training session took about 75 min including introduction, explanation of the procedures and performing DIBH and PBH from inhalation (PIBH) [21]. The second session, during which the breathing control strategies were repeated and RB at 22 brpm and PBH from exhalation (PEBH) were added, usually took not more than 45 min. In two subsequent sessions on a 3 T MRI (Ingenia, Philips Healthcare), imaging was performed during the breathing control strategies. These sessions (MRI1 and MRI2) took about 90 min each including preparation and set-up. The field of view included the lungs (partially) and upper abdomen to enable measurement of right diaphragm dome motion during the various breathing control strategies. All equipment was MRI-safe at 3 T.

Due to logistical reasons, time intervals between training sessions varied from a day to a week, and were at least one week between MRI sessions.

### Breathing control strategies

Using MR imaging, the following breathing control strategies were investigated and compared to FB: 1. five sequential DIBHs of fifteen seconds to investigate the reproducibility of the right diaphragm dome position. Volunteers were asked to hold their breath after comfortable inhalation; 2. RB at 22 brpm and reduced inflation volume; and 3. single PIBH and PEBH during hypocapnia. Hypocapnia was not induced for the other motion control strategies.

### MRI acquisition

Each breathing control strategy required a different MRI acquisition as listed in Table 1. During DIBH, the upper abdomen was imaged using a fast balanced turbo field echo (BTFE) 3D MRI acquisition in fourteen seconds to obtain one snapshot of the anatomy during each breath-hold. To visualize the right diaphragm dome’s respiratory motion amplitude during FB and RB, 4DMRIs were acquired using a T2-weighted turbo spin echo multi-slice 2D scan as reported by van Kesteren et al. [35]. In short, prior to each coronal slice acquisition, the position of the right diaphragm dome was determined using a 1D navigator. During a six minute acquisition, 60 volumes (dynamics) were scanned. The 2D slices were sorted based on the navigator position into ten amplitude bins after outlier rejection (discarding images corresponding to the 5% outmost navigator positions). This resulted in ten respiratory correlated 3D images depicting the anatomy during the various phases of the respiratory cycle. A 3D cine-MRI acquisition consisting of a sequence of 3D images with a temporal resolution of eleven to fourteen seconds was used for PBH.

**Table 1:** Overview of MRI acquisitions and scanning parameters

Breathing control strategy		DIBH	FB/RB	PBH
MRI strategy		3D MRI	4D MRI	3D cine-MRI
Sequence type		BTFE	TSE	BTFE
Resolution	(mm^3^)	2.0 × 2.0 × 2.0	1.3 × 1.6 × 4.0	1.6 × 1.6 × 1.6
Field of view	(mm^3^)	450 × 400 × 240	400 × 306 × 49	450 × 400 × 240—280
Echo time	(ms)	1.36	50	1.26
Repetition time	(ms)	2.7	562	2.5
Flip angle	(deg)	20	90	20
Slice orientation		Coronal	Coronal	Coronal
Dynamic time	(s)	14	6.2	11.4–14.5
Water-fat shift	(pixels)	0.266	0.658	0.389

For each breathing control strategy a dedicated MRI sequence was used. Dynamic time is the scan time needed to acquire one 3D image. The field of view had to be expanded depending on each volunteer’s anatomy for the 3D cine-MRI acquisition; therefore the dynamic scan time varies between volunteers. Abbreviations: deep inspiration breath-holds (DIBH), free breathing (FB), regularized breathing (RB), prolonged breath-holding (PBH), Balanced Turbo Field Echo (BTFE), Turbo Spin Echo (TSE)

### Motion quantification

**Fig. 2:**
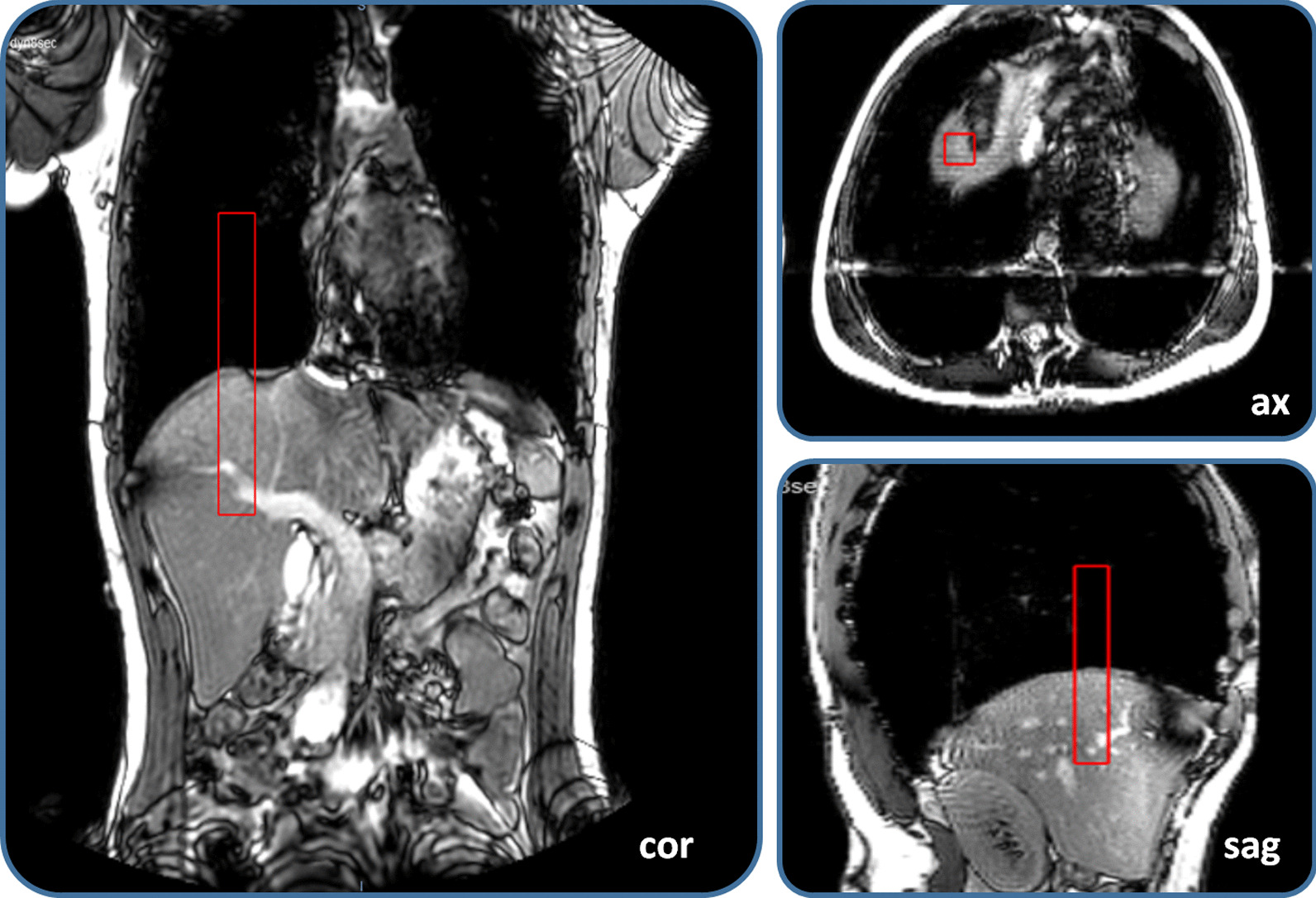
Diaphragm displacements were determined by translation in cranial-caudal direction aligning a region of interest (ROI) on the most cranial part of the right diaphragm dome at the anterior–posterior level of the pancreas. The ROI (fixed size, 2 × 2x15 cm) is depicted by the red box, shown in coronal (cor), axial (ax) and sagittal (sag) view of a 3DMRI

Displacement of the diaphragm between time points was quantified by translation in cranial-caudal direction of the various images (Velocity®, R4.0, Varian Medical Systems). First, the bony anatomy (spinal column) was automatically registered to correct for possible volunteer’s displacements between image acquisitions. Second, a manual translation in cranial-caudal direction was done on the most cranial part of the right diaphragm dome at the level of the pancreas in anterior–posterior direction, see Fig. 2.

**Fig. 3:**
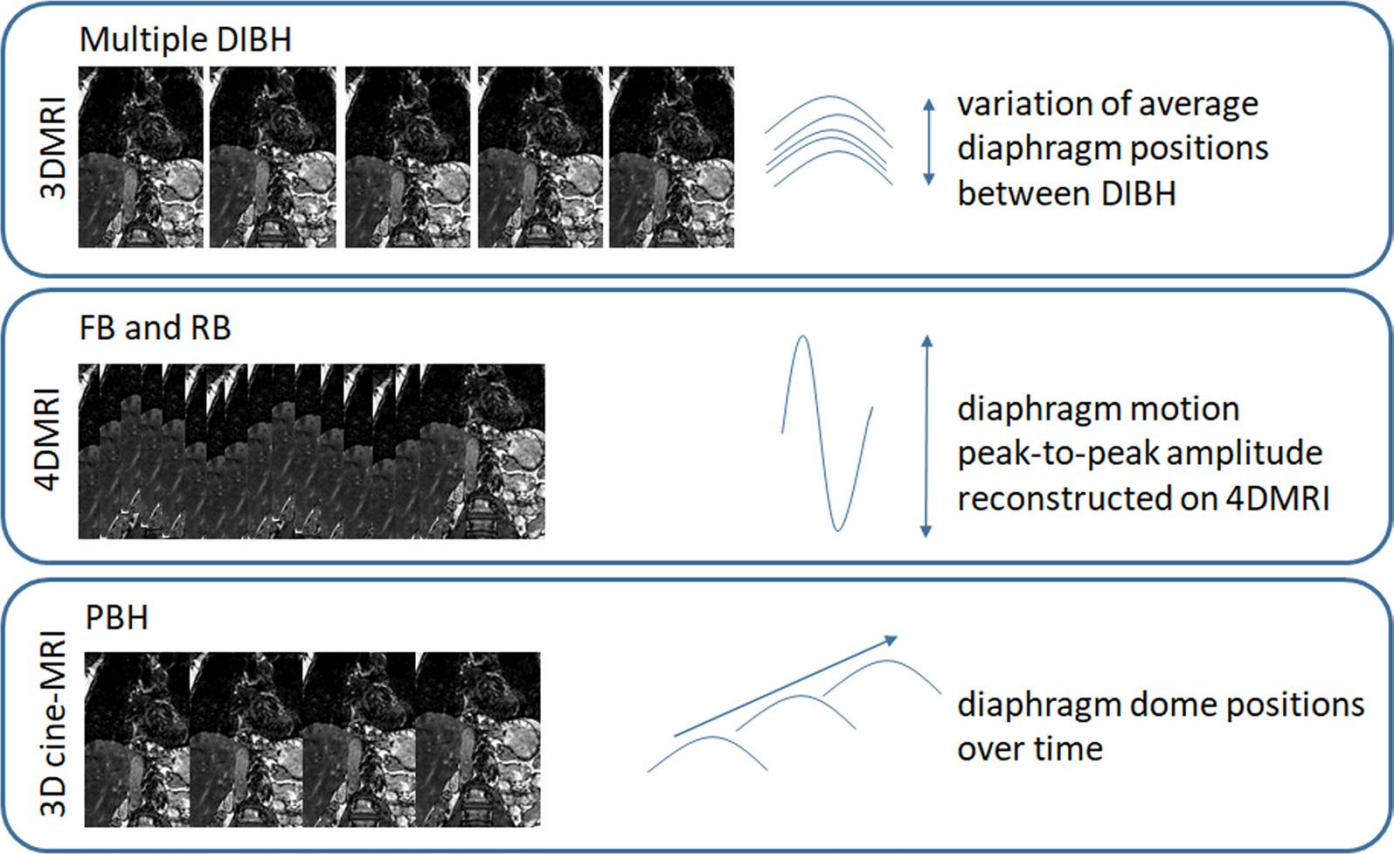
Schematic overview of the diaphragm motion metrics. For DIBH the variation of average diaphragm positions during the five consecutive breath-holds was measured. For FB and RB, the diaphragm motion peak-to-peak amplitude was determined. For PBHs, the diaphragm dome positions over time were measured. Abbreviations: deep inspiration breath-holds (DIBH), free breathing (FB), regularized breathing (RB), prolonged breath-holding (PBH)

Figure 3 shows an overview of how diaphragm motion during the various breathing control strategies was quantified. The variation in diaphragm position between five consecutive DIBHs per volunteer was expressed as inter-quartile range (IQR) of each average diaphragm position in the five breath-holds. During FB and RB, we determined the diaphragm motion amplitude on the 4DMRIs. The respiratory correlated 3D images were co-registered with respect to one reference image (e.g. the end-exhalation bin), resulting in diaphragm displacements reflecting the breathing motion. The largest diaphragm motion was taken as the breathing motion (peak-to-peak) amplitude. To determine the FB and RB motion amplitude reproducibility, we calculated the differences in motion amplitudes between sessions.

During PBH, the diaphragm dome positions over time were determined. Six dynamics were registered against the first dynamic (the reference image), i.e. three dynamics at 25%, 50% and 75% of the total PBH duration, and the last three at the end of the PBH duration. Displacements were linearly fitted to estimate the velocity of the diaphragm drift (mm/minute).

### Statistical analysis

Paired comparisons as appropriate were made for each volunteer between FB and RB, PIBH and PEBH and between MRI1 and MRI2 for all breathing control strategies. Using the Shapiro Wilk’s test combined with Q-Q plots we concluded that the data was not normally distributed. Hence, we used the non-parametric Wilcoxon signed rank test to determine significance of differences between measurements (SPSS Statistics Version 26, IBM, Armonk NY) and the Levene’s test to verify equivalence of variances [36]. A two-tailed *p*-value of < 0.05 was considered statistically significant. Data is expressed as median values with ranges and box plots as described.

## Results

Three volunteers dropped out after the second training session; two because of communication issues and one for health reasons unrelated to the study interventions. RB and PBH were well tolerated by the remaining fifteen volunteers available for analyses (V1-V15; 8 M/7F) having a median age of 22 years (mean 34; range 21–62) years. In six cases PBH during MRI was terminated when SpO2 fell < 94%. In one case a PBH was terminated because the volunteer had safely held for over 10 min (with SpO2 = 97% and normal blood pressure). For Volunteer 5, part of the data was lost due to data corruption (MRI navigator data was not available). One 4DMRI dataset could not be analyzed due to banding artefacts at the level of the diaphragm.

### Multiple deep inspiration breath-holds (DIBH)

**Fig. 4:**
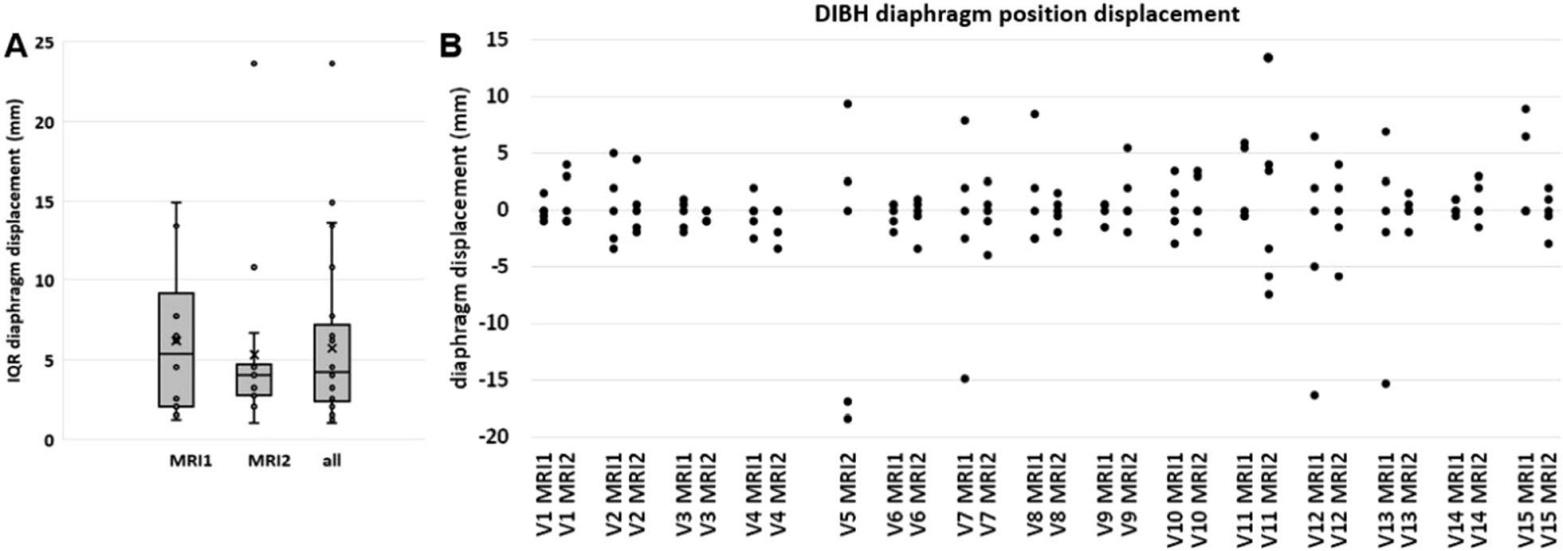
Diaphragm position variation in consecutive Deep Inspiration Breath-holds (DIBH). **A** Boxplots of inter-quartile ranges (IQR) of the diaphragm displacements of MRI1, MRI2 and both MRI sessions pooled. Diaphragm displacement variation did not significantly differ between MRI sessions MRI1 and MRI2. Boxes: median value and lower and higher quartiles, whiskers: lowest and highest data point within 1.5 times the inter-quartile range, ‘x’ denotes the mean value. **B** Diaphragm displacements with respect to the median DIBH diaphragm position during five consecutive DIBHs in two MRI sessions (MRI1 and MRI2) per volunteer. Note that for V11 in MRI2, six DIBH scans were available and analyzed

The displacement of the diaphragm dome within each of the five consecutive DIBHs is shown in Fig. 4. The median IQR of the diaphragm positions over the five acquisitions of all volunteers and all sessions was 4.2 (1.0–23.6) mm. Considering the whole group and both MRI sessions, 90% of the diaphragm displacements were within 12 mm. The median IQR diaphragm displacement over all volunteers was 5.4 (1.2–14.9) mm for session MRI1 and 4.0 (1.0–23.6) mm for session MRI2 (Fig. 4B). Between sessions, the IQR within volunteers did not differ significantly.

### Free breathing and regularized breathing

**Fig. 5:**
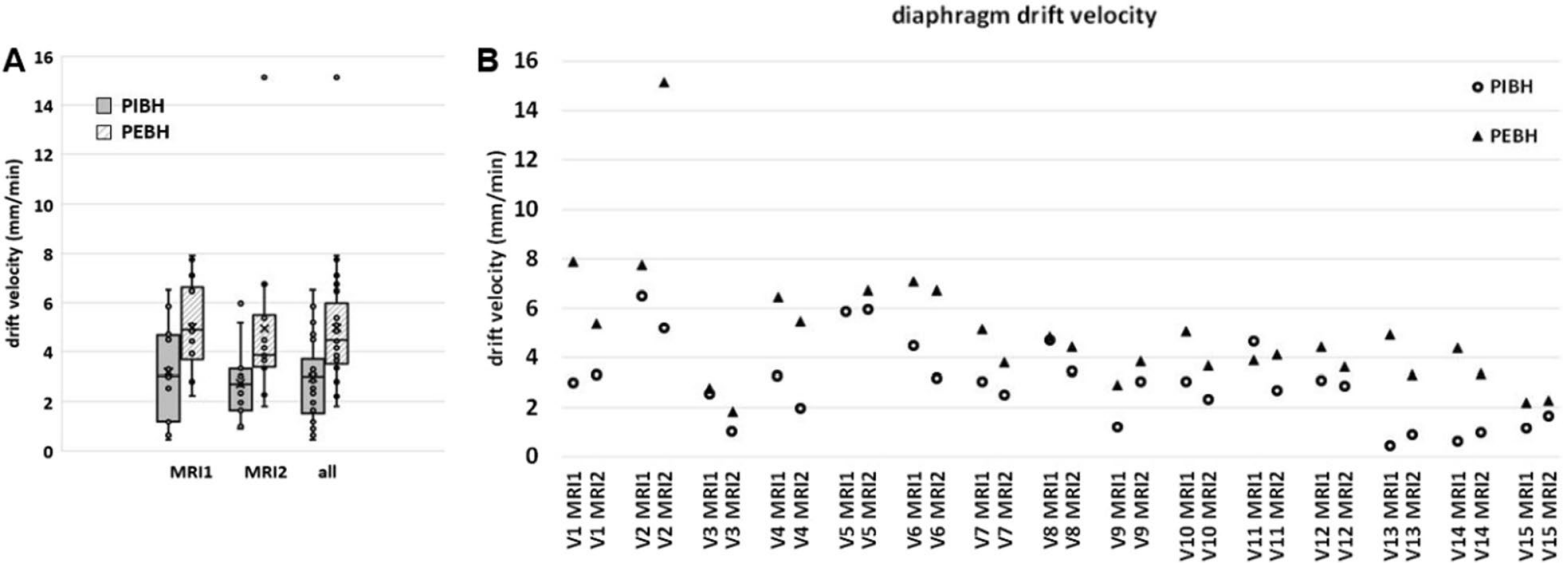
Regularized breathing (RB) significantly reduces diaphragm motion compared to free breathing (FB). Breathing peak-to-peak amplitude of the right diaphragm excursion in cranio-caudal direction, shown **A** over all volunteers per session, and **B** per volunteer and session. Regularized breathing at 22 brpm (triangles) induced by non-invasive mechanical ventilation demonstrated significantly smaller amplitudes compared to free breathing (FB, circles) in both MRI sessions. Boxes: median value and lower and higher quartiles, whiskers: lowest and highest data point within 1.5 times the inter-quartile range, ‘x’ denotes the mean value

Two out of sixty breathing motion amplitudes could not be reconstructed from the 4DMRIs acquired during FB and RB due to a missing acquisition or due to banding artefacts on the level of the diaphragm. The median reconstructed peak-to-peak amplitude was 39% (− 20–77%) smaller (*p* < 0.001) during RB: 12.8 (6.2–23.8) mm compared to FB: 20.9 (10.6–41.9) mm when pooling MRI1 and MRI2 (Fig. 5).

The median difference in breathing motion amplitude between MRI sessions (i.e., amplitude MRI1 minus amplitude MRI2) was − 2.2 (− 13.2–10.3) mm for FB, and − 2.4 (− 13.6–3.7) mm for RB. For FB the median amplitudes in sessions MRI1 and MRI2 were not significantly different, for RB the median amplitudes were significantly larger (*p* = 0.024) for MRI2. The median relative variation, calculated as the absolute amplitude difference (MRI1-MRI2)/(average amplitude MRI1-MRI2) was 16% and 18% for FB and RB, respectively.

### Prolonged breath-holding

The median duration of PIBH over both sessions was 7.1 (2.0–11.1) minutes and 5.8 (1.8–10.2) for PEBH (*p* = 0.001), see Table 2. PIBH and PEBH durations did not differ significantly between the two MRI sessions.

**Table 2:** Breath-hold durations for PIBH and PEBH during sessions MRI1 and MRI2

Volunteer	MRI1		MRI2	
	Duration (min)		Duration (min)	
	PIBH	PEBH	PIBH	PEBH
V1	7.0	4.4	7.5	6.1
V2	2.0	2.0	3.3	1.8
V3	4.3	3.6	4.1	4.2
V4	5.3	4.8	4.9	4.7
V5	7.1	6.2	8.0	6.4
V6	7.1	6.0	5.3	5.0
V7	7.7	6.2	7.1	5.3
V8	7.4	7.9	6.9	7.8
V9	4.8	7.1	6.3	5.3
V10	6.1	5.5	5.5	5.6
V11	7.3	7.6	9.3	8.4
V12	7.4	5.0	8.4	7.6
V13	5.3	3.9	5.0	5.2
V14	8.1	7.0	10.6	8.1
V15	11.5	10.2	11.1	8.6
Median	7.1	6.0	6.9	5.6
Min	2.0	2.0	3.3	1.8
Max	11.5	10.2	11.1	8.6

*PIBH*: prolonged inspiration breath-holding,* PEBH*: prolonged expiration breath-holding

**Fig. 6:**
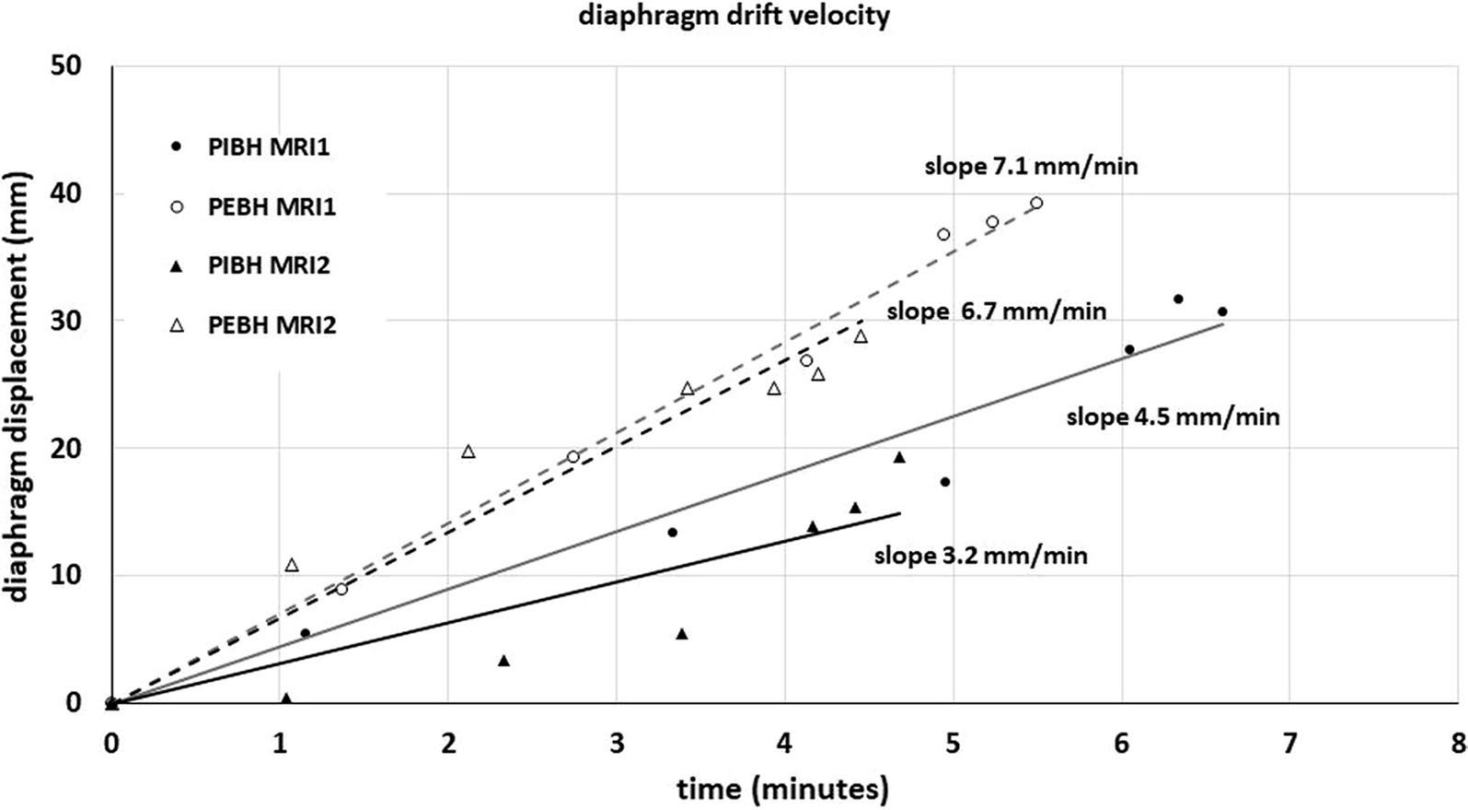
Example of the calculation of drift velocity during prolonged breath-hold (PBH). The diaphragm displacement during a PBH is measured at six time points, shown for volunteer 6. The drift velocity is determined by the slope of the linear fit (solid: PIBH, dashed: PEBH) through the measurements with diaphragm displacement in CC direction (mm) versus time (minutes). This example shows that drift velocity during PBH from inhalation (PIBH) is smaller than during PBH from exhalation (PEBH) for this volunteer

**Fig. 7:**
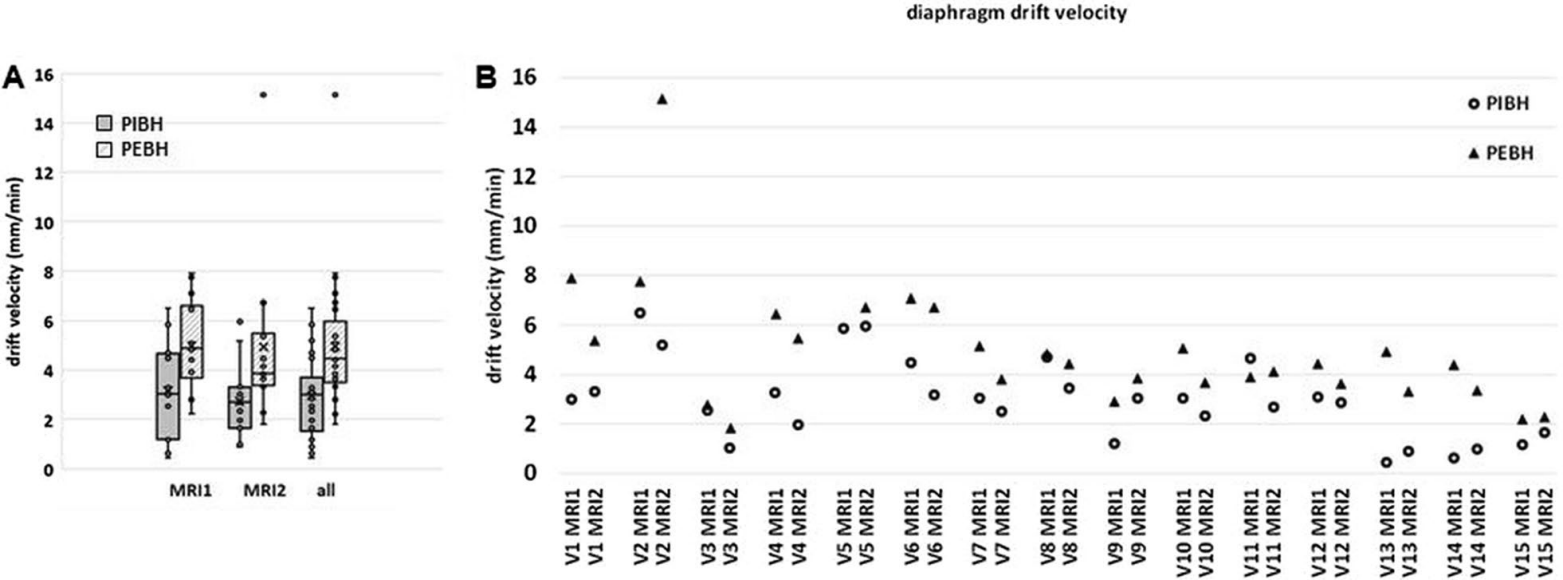
Cranial drift of the diaphragm dome during PIBH and PEBH. The diaphragm drift velocity during PIBH and PEBH **A** over all volunteers per session and **B** per volunteer and session are depicted, showing that the drift velocity is smaller during PIBH than during PEBH. Boxes: median value and lower and higher quartiles, whiskers: lowest and highest data point within 1.5 times the inter-quartile range (IQR), dots outside the whiskers: outliers, data points outside 3 times the IQR, ‘x’ denotes the mean value

Figure 6 shows the diaphragm displacement during the PBH duration. A cranial drift was observed for all PBHs for all volunteers. The average PBH drift velocity was determined from a linear fit of six diaphragm displacements. Figure 7 shows the drift velocities during both PIBH and PEBH in sessions MRI1 and MRI2 for all volunteers. Diaphragm drift velocities between sessions MRI1 and MRI2 did not differ significantly, so data from both was pooled. During PIBH the median diaphragm drift velocities was 3.0 (0.4–6.5) mm/minute. This was significantly smaller than that during PEBH 4.4 (1.8–15.1) mm/minute (*p* < 0.001).

A summary of the medians and ranges of diaphragm motion for DIBH, FB, RB and PBH for both MRI sessions is presented in Table 3.

**Table 3:** Overview of diaphragm motion during the breathing control strategies under investigation

Median (range) values	MRI1	MRI2	N	*p*-value
Testing differences between MRI sessions				
DIBH IQR (mm)	5.4 (1.2–14.9)	4.0 (1.0–23.6)	14	0.221
FB amplitude (mm)	20.2 (10.6–31.9)	22.1 (12.8–41.9)	14	0.362
RB amplitude (mm)	11.3 (6.2–20.3)	16.6 (9.3–23.8)	14	**0.024**
PIBH drift (mm/minute)	3.0 (0.4–6.5)	2.7 ( 0.9–6.0)	15	0.125
PEBH drift (mm/minute)	4.9 (2.2–7.9)	3.8 (1.8–15.1)	14	0.064

Median (range) values	FB	RB	N	*p*-value
Testing differences between breathing control strategies				
MRI1 amplitude (mm)	20.2 (10.6–31.9)	11.3 (6.2–20.3)	14	**< 0.001**
MRI2 amplitude (mm)	22.1 (12.8–41.9)	16.6 (9.3–23.8)	14	**0.002**
MRI1 & MRI2 amplitude (mm)	20.9 (10.6–41.9)	12.8 (6.2–23.8)	28	**< 0.001**

Median (range) values	PIBH	PEBH	N	*p*-value
Testing differences between breathing control strategies				
MRI1 drift (mm/minute)	3.0 (0.4–6.5)	4.9 (2.2–7.9)	14	**0.002**
MRI2 drift (mm/minute)	2.7 ( 0.9–6.0)	3.8 (1.8–15.1)	15	**0.001**
MRI1 & MRI2 drift (mm/minute)	3.0 (0.4–6.5)	4.4 (1.8–15.1)	29	**< 0.001**

Bold values indicate the significant* p*-values

For deep inspiration breath-holds (DIBHs), diaphragm motion is expressed by the inter-quartile range (IQR) of the consecutive breath-holds. For free breathing (FB) and regularized breathing (RB) at 22 breaths per minute the diaphragm motion is quantified by the breathing motion amplitude measured on 4DMRIs. For prolonged breath-hold from inhalation (PIBH) and exhalation (PEBH), diaphragm motion is expressed in the diaphragm drift velocity. Due to missing data and unsuccessful reconstructions, the number of pairs to be compared (N) per test varies. Significance was tested with a Wilcoxon signed-rank test (α = 0.05). *P*-value is shown in boldface when the difference was significant

## Discussion

In this study we quantified the motion magnitude of the right diaphragm dome during free breathing and DIBH, and various breathing control strategies supported by non-invasive mechanical ventilation. We investigated such motion control strategies using repeated MR imaging in each of the healthy volunteers. Regularized breathing and prolonged breath-holds prepared with preoxygenation and mechanically induced hypocapnia were investigated as possible alternatives for short DIBHs with air (without any preparation), being the current clinically used standard besides free breathing to minimize organ motion during radiotherapy.

In fifteen healthy volunteers we first assessed the variation of the right diaphragm dome position during multiple repeated DIBHs. Secondly, we investigated the diaphragm motion during RB at 22 brpm compared to free breathing. Finally, we analyzed residual diaphragm motion during PBHs from inhalation and exhalation on MRI.

The median IQR of the diaphragm positions over the five DIBH acquisitions of all volunteers and all sessions in our study was 4.2 mm, which is similar to interfraction variabilities reported in literature [15, 37]. In a study of fifteen pancreatic cancer patients, the diaphragm position variation in cranio-caudal direction between multiple DIBHs were found to have a group mean of 0.5 (SD 2.9) mm as measured on fluoroscopic [15]. This is in line with the 3.3 mm SD between successive breath-holds found during lung stereotactic body radiation therapy (SBRT) using breath-holding assisted with spirometry and repeat CT imaging [37]. Assuming a normal distribution and converting IQR = SD/1.35, these numbers are comparable to what we found in our study. Furthermore, we observed diaphragm displacement variations between DIBHs of up to 23.6 mm, in line with up to 19.9 mm reported by Lens et al. for pancreatic cancer treatment [15]. In patients treated with liver SBRT, it was shown that variations in daily breath-holding can have a large effect on interfractional diaphragm positions with respect to the vertebrae position varying from − 14 to + 15 mm [38]. his implies a risk of tumor misses when treating patients with repeated DIBHs. Finally, it should be noted that *within* a DIBH of 60 s the diaphragm drifts in the cranial direction. Holland et al. observed diaphragm drifts of up to 0.6 mm/s during DIBH, and Lens et al. showed that the diaphragm may move about 10 mm in cranio-caudal direction within one minute, from which 3.2 mm motion takes place in the first 10 s of the DIBH [14, 16].

MRI has been used previously to demonstrate how rapid shallow breathing with mechanical ventilation reduces breathing amplitudes with respect to FB [22, 24, 39]. Our mechanically induced rapid shallow breathing at 22 brpm, also resulted in significantly smaller peak-to-peak amplitudes compared to FB. We measured amplitudes of 11.3 mm and 16.6 mm in the respective two MRI sessions, which is comparable with the mean respiratory amplitudes of 9.4 mm and 10.5 mm at 20 brpm, and 8.0 mm and 8.6 at 25 brpm, respectively as measured on MRI in ten healthy volunteers [24]. Furthermore, in that study mean amplitude reductions of 56% and 62% for 20 and 25 brpm, respectively were reported. Van Ooteghem et al. analyzed shallow-controlled breathing at 30 brpm showing mean amplitude reductions of 36% compared to volume-controlled breathing, and 4% compared to spontaneous breathing [23]. We found a median relative amplitude reduction of 39% during RB at a frequency of 22 brpm compared to FB.

Previously, single PBH from inhalation (> 5 min) has been demonstrated to be feasible in healthy volunteers and in breast cancer patients [20–22, 34]. However, as MRI data evaluating internal motion during PBHs was not available up to now, our study is the first to quantify residual diaphragm motion during PBHs from inhalation and exhalation using MRI. Conform another report we demonstrated a displacement of the right diaphragm dome in cranial direction during breath-holding [16]. The cranial displacement of the diaphragm is a consequence of the gradual lung deflation caused by gas exchange in the lungs, whereby the uptake of oxygen from the lungs to the blood is not equally compensated by the secretion of carbon dioxide from the blood to the lungs [16, 19]. The linearly fitted displacements of the right diaphragm dome over time showed the median diaphragm drift velocity to be smaller during PIBH (3.0 mm/minute) than during PEBH (4.4 mm/minute). We argue that this is due to the same volume of oxygen being extracted having a greater proportional effect on lung volume at initially smaller lung volumes. In contrast, mean diaphragm motion velocities during DIBH (i.e. 20 s)—also measured on MRI, were reported to be greater during end-inspiration (~ 0.6 mm/s) than during end-expiration (0.15 mm/s) breath-holding [16]. Similar results were reported in a study comparing diaphragm motion magnitude and velocity during 60 s breath-holds with different lung volumes where the motion magnitude in cranial direction was larger during inhalation breath-holds than during exhalation breath-holds [15].

In our study we focused on the quantification of diaphragm motion since this possibly is the structure in the abdomen that moves the most. We limited our measurements to the motion of the top of the right diaphragm dome in cranial-caudal direction only by translations at the level of the pancreas in anterior–posterior direction. Since the curvature of the diaphragm will be different at different lung inflation levels, this introduces additional uncertainties. However, as in previous work [14], we found that the ventral and dorsal region of the diaphragm move differently than the mid diaphragm, suggesting that deformable image registration techniques might yield higher accuracy. Whereas the diaphragm motion is highly correlated with liver motion, other abdominal organs including spleen, pancreas and kidneys might move differently and/or to a lesser extent, and the diaphragm might not be a direct surrogate for abdominal organ (and tumor) motion [15, 40].

Using the investigated breathing control strategies in radiotherapy potentially reduces radiation-associated toxicities by decreasing the margins around the target volume, and sparing healthy tissues. Regularized breathing with mechanical ventilation at 22 brpm reduced the median motion amplitude from 20 to 12.4 mm in our cohort. This would correspond with an ITV reduction of 7.6 mm in cranial-caudal direction. When considering a mid-position approach as investigated by Lens et al., the PTV would be reduced from around 15–11 mm utilizing RB for lung cancer treatment, and 17–13 mm for pancreatic cancer treatment [3].

A limitation of this study is that the healthy volunteers were relatively young (median age 22 years). However, it has been shown that RB is well tolerated by lung, liver and breast cancer patients up to 83 years old and PBH is well tolerated in breast cancer patients up to 74 years old [21, 41]. We therefore do not expect important difficulties when we include patients in clinical studies.

In radiotherapy, typically DIBH durations of 30 s are used. Considering the intra-DIBH diaphragm drift of 3.2 mm displacement in the first 10 s, and 2.8 mm displacement in the following 20 s, this accounts for a 6 mm displacement within a DIBH which is not incorporated in safety margins. On top of this, consecutive voluntary DIBHs vary with regard to volume and amplitude, with a 4 mm IQR variation in our volunteer cohort. At the treatment machine, the inter-DIBH variation can be reduced with the aid of breath-holding tools such as a spirometer with visual feedback to increase lung volume reproducibility, or an active breath-holding control system, which was not available at our MRI experiments. Based on our results, incorporating both inter-DIBH variation and intra-DIBH motion into a margin recipe is not straightforward and requires more research. Furthermore, a PIBH of 10 min would require a 3 cm margin to incorporate the steady drift, which is highly unfavorable. Tumor tracking during PIBH at the linac would be one viable approach to account for this motion. Alternatively, we are investigating how to compensate for the gradual lung deflation during PBH with gradual lung re-inflation.

## Conclusion

Regularized breathing at a frequency of 22 breaths per minute resulted in significantly smaller peak-to-peak breathing amplitudes compared to free breathing. Furthermore, prolonged breath-holding from inhalation and exhalation with median durations of six to seven minutes are feasible. Prolonged breath-holding may be a promising breathing control strategy when compensation for the gradual lung deflation can be achieved.

## Data Availability

The datasets generated and/or analyzed during the current study are not publicly available since the volunteers did not consent in sharing the data with third parties.

## Abbreviations

4DCT: Four-dimensional computed tomography
4DMRI: Four-dimensional magnetic resonance imaging
Ax: Axial
brpm: Breaths per minute
BTFE: Balanced turbo field echo
CO_2_: Carbon dioxide
Cor: Coronal
CTV: Clinical target volume
DIBH: Deep inspiration breath-hold
FB: Free breathing
GTV: Gross tumor volume
IQR: Inter-quartile range
ITV: Internal target volume
MRI: Magnetic resonance imaging
O_2_: Oxygen
PBH: Prolonged breath-hold
PETCO_2_: Partial end-tidal pressure of carbon dioxide
PEBH: Prolonged breath-hold in exhalation after a short sigh
PIBH: Prolonged breath-hold in end inhalation
RB: Regularized breathing
PTV: Planning target volume
ROI: Region of interest
Sag: Sagittal
SBH: Short breath-hold
sBP: Systolic blood pressure
SBRT: Stereotactic body radiation therapy
SpO_2_: Oxygen saturation
T2W: T2-weighted
TSE: Turbo spin echo
