# Correction: Quantifying the reduction of respiratory motion by mechanical ventilation with MRI for radiotherapy

**DOI:** 10.1186/s13014-022-02071-w

**Published:** 2022-06-28

**Authors:** Z. van Kesteren, J. K. Veldman, M. J. Parkes, M. F. Stevens, P. Balasupramaniam, J. G. van den Aardweg, G. van Tienhoven, A. Bel, I. W. E. M. van Dijk

**Affiliations:** 1 Department of Radiation Oncology, Amsterdam UMC Location University of Amsterdam, Meibergdreef 9, Amsterdam, The Netherlands; 2 Department of Anesthesiology, Amsterdam UMC Location University of Amsterdam, Meibergdreef 9, Amsterdam, The Netherlands; 3 Department of Anesthesiology, Amsterdam UMC Location Vrije Universiteit Amsterdam, De Boelelaan 1117, Amsterdam, The Netherlands; 4 Department of Pulmonology, Amsterdam UMC Location University of Amsterdam, Meibergdreef 9, Amsterdam, The Netherlands

## Correction to: Radiation Oncology (2022) 17:99 10.1186/s13014-022-02068-5

After publication of this article [1], the authors reported that a wrong figure appeared as Fig. 5; the figure should have appeared as shown below.

**Fig. 5:**
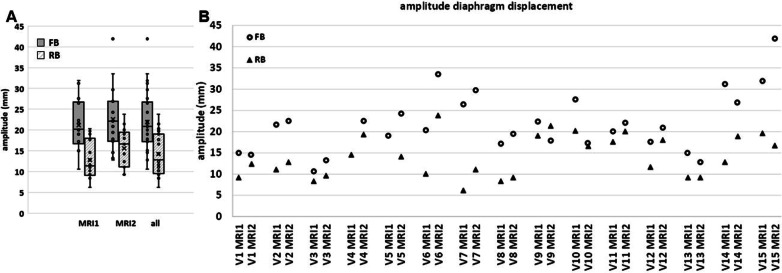
Regularized breathing (RB) significantly reduces diaphragm motion compared to free breathing (FB). Breathing peak-to-peak amplitude of the right diaphragm excursion in cranio-caudal direction, shown **A** over all volunteers per session, and **B** per volunteer and session. Regularized breathing at 22 brpm (triangles) induced by non-invasive mechanical ventilation demonstrated significantly smaller amplitudes compared to free breathing (FB, circles) in both MRI sessions. Boxes: median value and lower and higher quartiles, whiskers: lowest and highest data point within 1.5 times the inter-quartile range, ‘x’ denotes the mean value

The original article [1] has been updated.
